# Study protocol for an observational cohort study of heat stress impacts in pregnancy in The Gambia, West Africa

**DOI:** 10.12688/wellcomeopenres.23172.2

**Published:** 2025-02-07

**Authors:** Ana Bonell, Leonidas G. Ioannou, Abdul Sesay, Kris A. Murray, Bubacarr Bah, David Jeffries, Sophie E. Moore, Ana Vicero-Cabrera, Neil S Maxwell, Jane E Hirst, Cally Tan, Apolline Saucy, Dorothy Watters, Bakary Sonko, Emmanuel Okoh, Yahaya Idris, Williams Oluwatosin Adefila, Jarra Manneh, Mam Leigh-Nabou, Sainabou Bojang, Andreas Flouris, Andy Haines, Andrew Prentice, Amanda N Sferruzzi-Perri

**Affiliations:** 1 Medical Research Council Unit The Gambia at the London School of Hygiene and Tropical Medicine, Banjul, Banjul, The Gambia; 2 Centre on Climate Change and Planetary Health, London School of Hygiene and Tropical Medicine Faculty of Epidemiology and Population Health, London, England, UK; 3 Department of Physical Education and Sport Science, FAME laboratory, Thessaly, Greece; 4 King's College London Department of Women & Children's Health, London, England, UK; 5 Institute of Social and Preventative Medicine, University of Bern, Bern, Switzerland; 6 University of Bern Oeschger Centre for Climate Change Research, Bern, Canton of Bern, Switzerland; 7 Environmental Extremes Laboratory, University of Brighton, Brighton, England, UK; 8 The George Institute for Global Health UK, Imperial College London, London, UK, Oxford, England, UK; 9 Centre for Trophoblast Research, University of Cambridge Department of Physiology Development and Neuroscience, Cambridge, England, UK

**Keywords:** heat, heat stress, pregnancy, fetal, infant, birth outcomes, physiology, placenta, climate change

## Abstract

Climate change has resulted in an increase in heat exposure globally. There is strong evidence that this increased heat stress is associated with poor maternal and fetal outcomes, especially in vulnerable populations. However, there remains poor understanding of the biological pathways and mechanisms involved in the impact of heat in pregnancy. This observational cohort study of 764 pregnant participants based in sub-Saharan Africa, a geographical region at risk of extreme heat events, aims to evaluate the physiological and biochemical changes that occur in pregnancy due to heat stress. The key objectives of the study are to 1) map exposure to heat stress in the cohort and understand what environmental, social and community factors increase the risk of extreme heat exposure; 2) assess the impact of heat stress on maternal health, e.g. heat strain, subjective psychological well-being, sleep and activity level; 3) evaluate how heat stress impacts placenta structure and function; 4) determine how chronic heat exposure impacts birth outcomes; and 5) explore the epigenetic changes in the placenta and infant by heat stress exposure per trimester.

Pregnant women will be recruited from two distinct regions in The Gambia to exploit the naturally occurring heat gradient across the country. Microclimate mapping of the area of recruitment will give detailed exposure measurements. Participants will be asked to wear a watch-style device at 28- and 35-weeks gestational age to evaluate maternal heart rate, activity and sleep. At the end of the week, an ultrasound scan will be performed to evaluate fetal size and placental blood flow. At delivery, birth outcomes will be recorded and maternal, placental and cord samples taken for epigenetic, biochemical and histological evaluation. Evaluation of neuro-behaviour and final infant samples will be taken at 1 month following birth.

## Plain language summary

Climate change has increased global temperatures. Women exposed to heat during their pregnancy have an increased risk of poor maternal and pregnancy outcomes including preterm birth and stillbirth. However, there is a poor understanding of the biological pathways that result in these poor outcomes when pregnant women are exposed to heat. This study will follow 764 pregnant women in two sites in The Gambia, West Africa - an area that is particularly at risk of heat waves and other extreme weather events. The study aims to understand or determine the following: the heat exposure pregnant women are being exposed to; the impact of heat stress on pregnant women; the impact of heat stress on the placenta; the impact of heat stress on birth outcomes; the epigenetic changes in the placenta and/or infant related to the heat stress exposure.

This observational study will recruit from two sites - cool coast and hot interior. Pregnant women will be closely followed up during the course of their pregnancy with regular ultrasound scans, questionnaires and both physiology and environmental monitoring. At delivery, birth data will be collected as well as samples from the placenta and cord blood. The final visit will be conducted at 1 month where the baby will be assessed for feeding, growth, sleep and neuro-behaviour and final samples taken.

## Introduction

Anthropogenic climate change has resulted in an increased risk of exposure to heat stress and extreme heat globally, but in particular to those people living in tropical and sub-tropical regions ^1^. Existing evidence shows that heat stress impacts both maternal and fetal health, with increased risk of pre-eclampsia ^2^, gestational diabetes ^3^, severe maternal morbidity ^4^, congenital abnormalities ^5^, miscarriage, stillbirth ^6^, preterm birth ^7^, low birth weight ^8^, and premature rupture of membranes ^9^, in those exposed to high heat stress versus no heat stress in pregnancy. Heat stress is defined as the environmental conditions (air temperature, humidity, solar radiation, wind speed) combined with the metabolic heat load and may result in heat strain, the physiological response to that heat stress and eventually in heat stroke, a potentially fatal condition. Although there is no consensus definition for extreme heat or heat waves, the most common definition is maximum temperatures exceeding the 95 ^th^ percentile for that region.

West Africa is a particularly vulnerable region to the impacts of climate change, with the compounding risks of extreme heat, food insecurity, vector-borne diseases, and water scarcity/flooding ^10^. In addition, half of agricultural workers are women, and many work during pregnancy ^11^. They perform much of the subsistence farm-work and many strenuous manual tasks throughout pregnancy. Previous studies have found that pregnant women struggle with physical symptoms, physiological heat strain, intensity of physical activity, and have few options for adaptation when working in the heat ^12, 13^. In addition, there is clear evidence that air pollution worsens the adverse effects of extreme heat and it is likely other environmental factors may also play a role ^14, 15^. 

Understanding the effects of heat may help determine the underlying physiological processes prior to trialling tailored interventions.

### Temperature regulation – considerations in pregnancy

Humans are homeothermic mammals and thermoregulation is one of the most basic homeostatic feedback loops, resulting in a core body temperature within a tightly controlled range around 37.0°C ^16, 17^. Heat balance is achieved by heat gain, from both internal (metabolic) and external (environmental) sources equalling heat losses. Thermal loss is dependent on both behavioural and physiological mechanisms. Behavioural change choices are extensive (such as seeking shade), although these may be limited in certain settings (e.g. occupational heat exposure), whereas physiological mechanisms to lose heat are limited to three; diversion of blood to the skin to increase radiative and convective heat loss, sweating to enable evaporative heat loss, and hyperventilation to increase respiratory heat loss ^17^. Maternal hyperthermia increases the risk of abnormal embryonic and fetal development ^18^. There are multiple physiological changes that occur during a human pregnancy that could affect thermoregulation. Cardiac output and plasma volume increase by up to 50% by the third trimester and dilutional anaemia occurs ^19^. It is thought that the increase in basal metabolic rate as pregnancy progresses, along with the decrease in body mass to surface area ratio both reduce the ability to loose heat. However this appears to be balanced by an overall lowering of the sweat threshold (therefore sweating occurs at lower environmental heat exposures) and by the decrease in core temperature that occurs as pregnancy progresses ^20^.

Although there remains some uncertainty as to the effectiveness of thermoregulation in pregnancy ^21^, recent studies have shown that thermal homeostasis is no more challenging in pregnancy than when not pregnant within usual environmental heat exposure ^20, 22, 23^. However, as discussed below there are several potential pathways implicated in the association between heat exposure and adverse birth outcomes, with maternal hyperthermia only being one of them.

### Potential pathophysiological pathways

**Figure 1.**
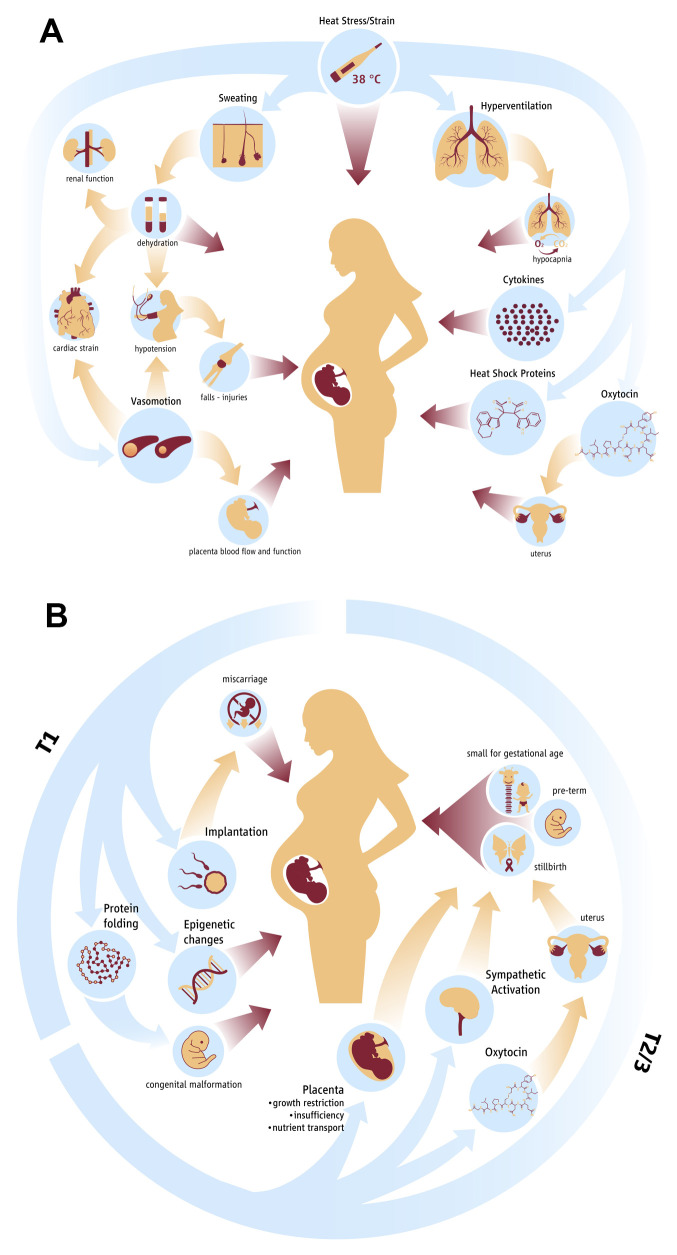
Hypothesised physiological pathways in acute ( **1A**) and chronic ( **1B**) heat stress exposure during pregnancy. **1A**: Acute effect of heat strain on fetal health and development. Blue arrows indicate the effects of heat strain on physiological mechanisms and biological markers. Yellow arrows indicate indirect effects of heat strain on physiology. Red arrows indicate direct effect of a parameter on fetal health and development. **1B**: Chronic effect of heat stress on maternal and fetal physiology and subsequent adverse outcomes.

Both short-term/acute (up to 4 weeks) and long-term/chronic (whole pregnancy) heat exposure adversely effects pregnancy outcomes and it is likely that the mechanistic pathways overlap but may not be exactly the same. Based on current available evidence in the literature ^8, 13, 18, 24– 28^, the pathways are visualized in Figure 1.

Despite these mechanisms being plausible from a theoretical and animal physiological understanding, detailed studies in humans are missing to provide definitive evidence ^20, 29^.

## Hypotheses

We hypothesize that pregnant women exposed to higher levels of ambient heat stress in pregnancy will experience higher levels of maternal heat strain, poorer sleep, changes in placental blood flow (detectable in vivo through alterations in the ultrasound Doppler of the umbilical arteries), structural and transcriptomic changes to the placenta and differences in birth and neonatal outcomes. We propose that there will be a differential impact depending on the gestational age at which exposure occurs. We hypothesize that pregnant women exposed to high heat stress in pregnancy will have detectable alterations in placental structure, gene expression and functional capacity. We also hypothesize that as pregnancy progresses, heat stress impacts on the maternal physiology will increase, as will the detrimental effects on sleep and well-being. These differential effects, if found, will enable gestational-age-specific intervention development to improve pregnancy outcomes for mother and child.

### Research aims

This project aims to determine the physiological and biochemical changes that occur in pregnancy due to heat stress and how these impact maternal, fetal and newborn health and well-being. The specific research questions and linked study objectives are outlined below.

### Research questions and objectives

***Exposure***

- What is the ambient heat stress exposure of pregnant women in coastal and inland Gambia?
  - *Derive high-resolution, spatiotemporal heat stress maps of the residential area of all study participants to determine heat stress exposure in pregnancy.*
- What factors influence the temporal and spatial distribution of heat stress?
  - *Identify areas in the study region most affected by heat stress and the potential relationship with geographic, topographic and ecological features, including proximity to water, vegetation and housing type.*
- Does air pollution and noise pollution exposure vary with heat exposure in urban and rural settings?
  - *Derive spatiotemporal maps of air pollution exposure through static outdoor devices and an ad hoc monitoring campaign of indoor air and noise pollution on a sub-set of participants.*

***Maternal impacts***

- What is the level of physiological heat strain experienced by pregnant women in coastal and inland Gambia throughout the year?
  - *Estimate physiological heat strain in pregnancy and how it varies by gestational age and other maternal, socio-economic and environmental factors.*
- What impact does ambient heat stress have on secondary outcomes (activity level, well-being, sleep quality, food security and food intake)?
  - *Determine the acute and chronic impact of heat stress on maternal activity level, well-being, sleep quality and food security, and how these vary with gestational age, maternal and socioeconomic factors.*
- How does acute and chronic heat stress exposure affect levels of maternal circulating placental hormone and inflammatory cytokine levels during pregnancy?
  - *Estimate changes in circulating placental hormone levels and inflammatory markers at 28 week gestation by measured heat stress and heat strain exposure.*

***Placental impacts***

- How does heat stress affect the materno-placental interface and what is the relationship between heat exposure and utero-placental blood flow and function?
  - *Determine the acute and chronic impact of heat stress on materno-placental blood flow, placental structure, vascular density and function.*
  - Which genes and pathways are affected in the placenta by heat stress exposure?
  - *Determine the differentially expressed genes and altered signalling pathways in the placenta in pregnancies exposed to differing levels of heat stress by trimester.*

***Birth and neonatal outcomes***

- How does maternal exposure to heat stress effect birth outcomes, such as small-for-gestational age, gestational-age-adjusted birth weight, gestational-age-adjusted birth length and gestational-age-adjusted head circumference, stillbirth and gestational length?
  - *Determine the effect of acute and chronic heat stress exposure by trimester on birth outcomes related to intra-uterine growth restriction.*
  - *Determine the effect of acute and chronic heat stress exposure on risk of stillbirth.*
  - *Determine the effect of acute and chronic heat stress exposure on gestational length.*
- How does maternal exposure to heat stress during pregnancy influence neonatal neuro-behavioural and neurological status?
  - *Determine whether maternal heat stress exposure by trimester is associated with differences in Neuro-Behavioural Assessment Scale (NBAS) assessment within 28 days of birth.*
- Which genes and pathways are altered in infant samples by heat stress exposure?
  - *Determine the differentially expressed genes and altered signalling pathways in infant blood samples (at 28 days) in pregnancies exposed to differing levels of heat stress by trimester.*

## Methods

### Study design

We will conduct a prospective observational cohort study of pregnant women who live in two contrasting regions of The Gambia - Brikama in West Coast Region (coastal region) and in and around Basse in Upper River Region (inland region).

**Figure 2.**
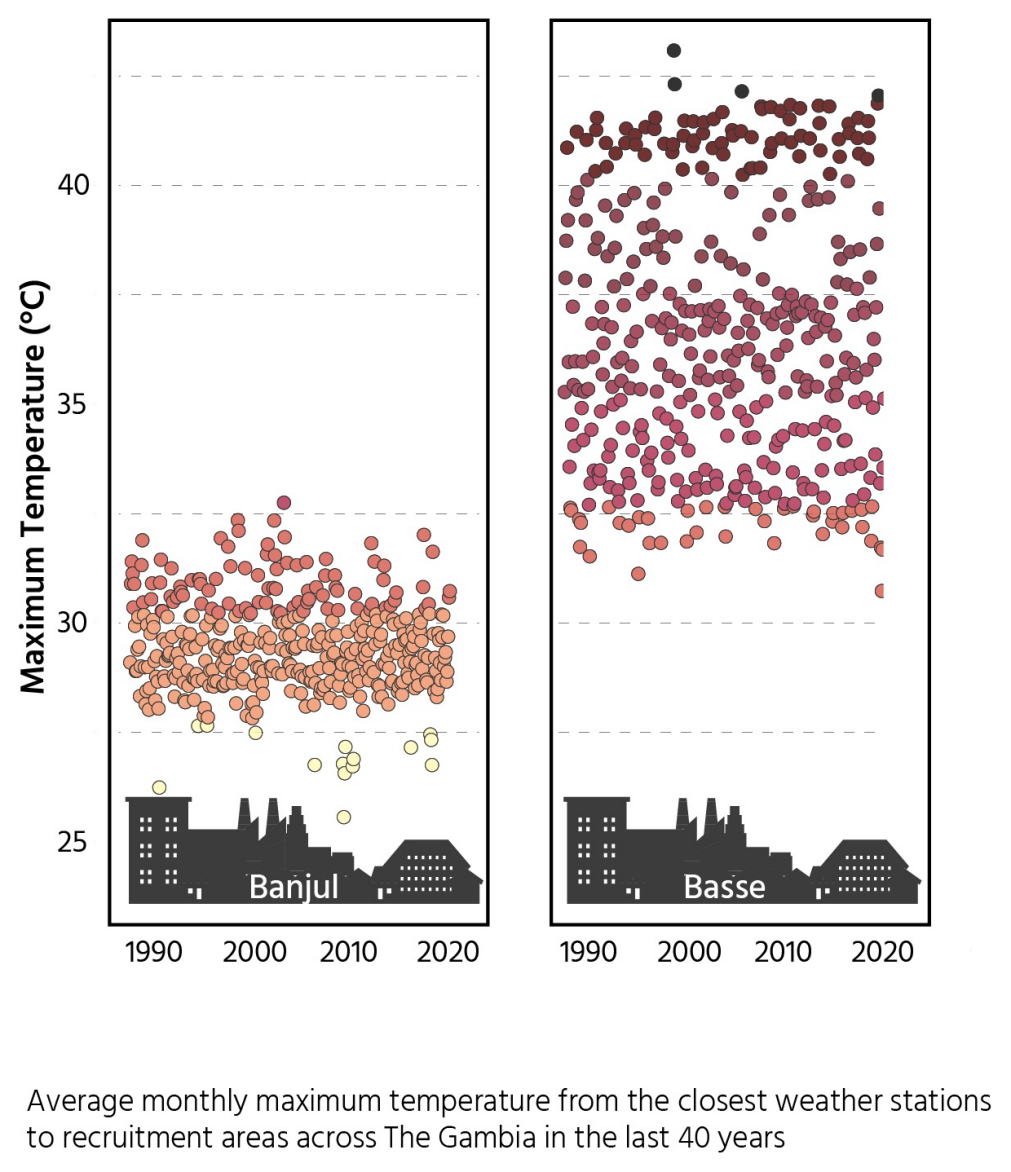
Maximum monthly temperatures for costal and inland regions.

This will utilize the temperature gradient that occurs within The Gambia as visualized in Figure 2 and include participants from both urban and rural areas. We will collect regular data on the main drivers of seasonal differences in birth outcomes in order to disentangle the impact of heat stress from other seasonal drivers, for example food security and infectious disease episodes which may both be climatically influenced.

Prior to recruitment of participants, we will set-up stationary heat stress meters (Kestrel® 5400 Heat Stress Monitor, produced by Nielsen-Kellerman Company, USA) that will act as weather stations for the study ^30, 31^. In addition, we will use stationary low-cost heat and humidity sensors (Kestrel Drop D2) to build a micro-climate map of the region the study participants live in. To ensure other potential environmental confounders are accounted for, we will also utilize stationary air pollution monitors (IQAir™) in the geographical region, as well as additional ad hoc monitoring to measure personalized noise and air pollution in a sub-set of participants (details below).

### Sample size

The study has multiple outcomes of interest related to heat stress exposure. Sample size calculations have been based on preliminary data in The Gambia or data from published research. However, certain outcomes have minimal data available to estimate an effect of heat (e.g. comparison of mean circulating placental hormones in those exposed to heat versus not exposed) and therefore we assume the largest estimate of sample size will be sufficient to detect meaningful differences in these outcomes. Please see Table 1 for full details.

**Table 1.** Summary of sample size calculations for key outcomes.

Outcome	Heat strain	Sleep	Birth outcome (any adverse pregnancy outcome)	Birth outcome (small for gestational age)
Model	Repeated measures ANOVA	Repeated measures ANOVA	Logistic regression	Linear regression
Number of groups	2	2	2	-
Number of measurements	240	10	-	-
Effect size	0.15	0.2	2.2 *	0.14
Predictors	-	-		5
Significance	0.05	0.05	0.05	0.05
Power	80%	80%	80%	80%
Number needed	702	396	324	661

*Assumed relative risk (based on Rekha *et al.* ^32^)

Due to fetal loss and loss to follow-up, we increased our final sample size to 764 to meet sample size requirements for all outcomes.

### Study population and selection criteria

Participants will be pregnant women who attend government-run health care facilities for antenatal care based at or out of Basse or Brikama hospitals. A community sensitization programme will be undertaken to discuss the study with the community leaders, community members, pregnant women, local healthcare workers and any interested members of the community.

Women will be identified at routine government-run antenatal clinics.

### Provision of written informed consent

Trained study staff members will undertake additional sensitisation and screening prior to undertaking written informed consent. Each potential participant will be addressed in the language of their choice and will undergo individual screening interviews, be given written information on the project and have time to ask questions and discuss with family members prior to taking informed consent. Informed consent will be taken in the presence of an impartial witness and if the participant is unable to write, her fingerprint will be used as substitute for a signature. Participants may withdraw consent at any time during the course of the study without any impact on future care provision.

Study staff will then screen the women and enrol them if all the criteria listed below are fulfilled. Gestational age will be confirmed by ultrasound scan using the V-scan air and based on crown-rump-length if <13 weeks gestation, or a combination of bi-parietal diameter, head circumference, abdominal circumference and femur length if between 13 weeks and 26 weeks gestation.

Pregnant women of 18 years and above, of any parity or gravida, will be eligible for enrolment. Participants will be recruited if they fulfil all of the following inclusion criteria:

1. Confirmed pregnancy with a single fetus.
2. Less than 26 weeks gestation (confirmed by ultrasound scan).
3. Live and work within the region and plan to stay in the region for the entire pregnancy and delivery.
4. Not currently in any condition that would require immediate medical attention, e.g. fever, abdominal pains, vaginal bleeding.
5. Willingness and ability to provide demographic and clinical information, clinical samples, including placental samples and wear a non-invasive portable device for continuous physiological monitoring.

Those diagnosed with HIV infection will be excluded.

### Study procedures

Routine antenatal care will be provided by the government-run health clinic as per standard treatment. Study-specific activities will be conducted by personnel hired for the purpose of the research study, and will include research clinicians, nurse/midwives, field workers, project manager, laboratory staff and data manager.

**Figure 3.**
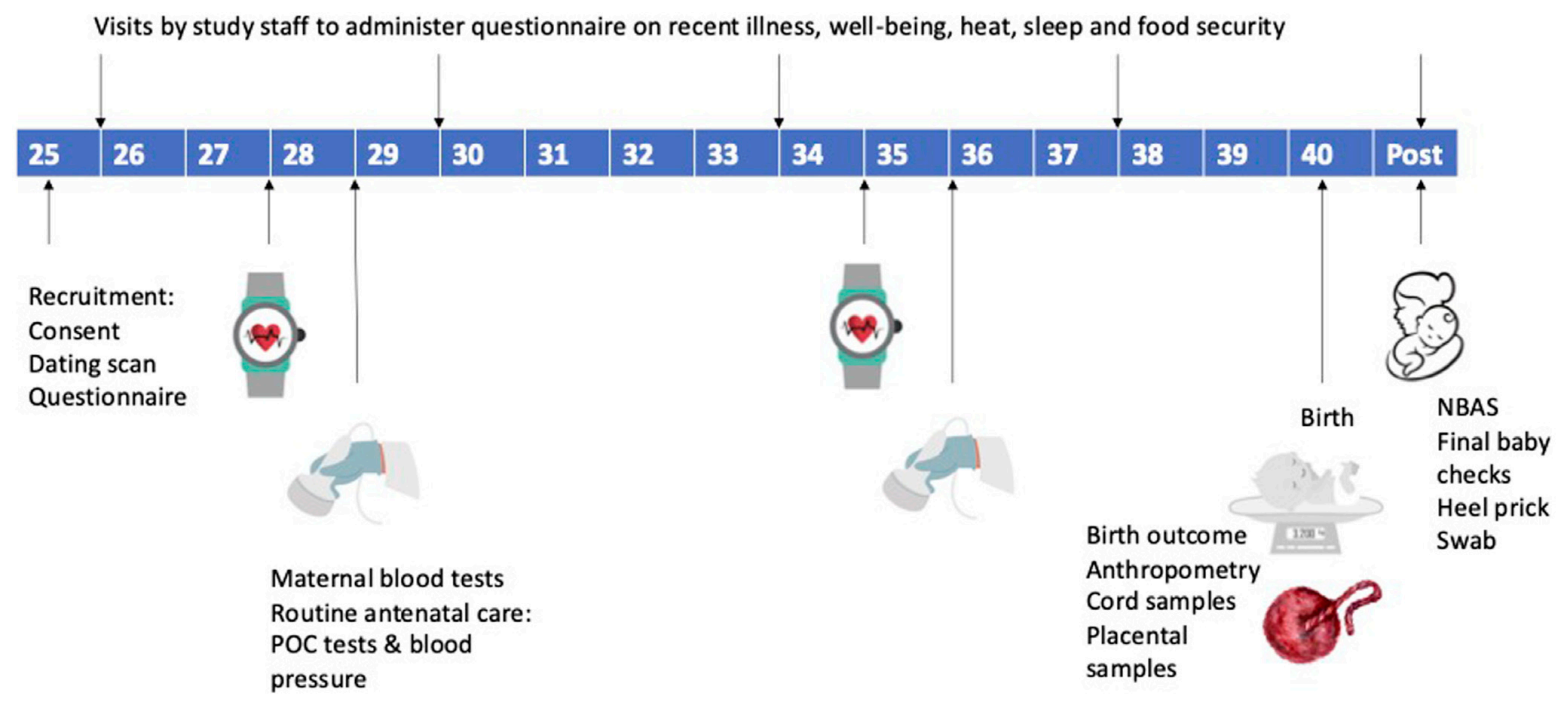
Study processes according to gestational age of participant pregnancy. POC = point-of-care tests (hemoglobin, blood glucose, urine analysis, dried blood spot for malaria PCR). NBAS = neonatal-behavioural assessment scale

Study staff will be trained in the protocol and relevant study procedures as visualised in Figure 3, prior to the start of the study. All approvals will be in place prior to commencement of the study and the study team will liaise closely with the hospital staff to ensure full communication within the facilities.

### Study activities and data collection at enrolment

During enrolment of each participant, study staff will collect basic demographic data (age, place of residence, type of housing, sanitation, cooking practices, noise annoyance, ethnicity, employment), obstetric history (including number of pregnancies, outcomes of those pregnancies, previous pregnancy related complications), current and past medical history (including disabilities), and current medication (including anti-malarial prophylaxis and micronutrient supplementation).

At the initial visit, study staff will also perform basic anthropometric measurements: height, weight and mid-upper arm circumference. A simple clinical examination will follow to include measurement of blood pressure with a digital device, heart rate, respiratory rate, tympanic temperature, and uterine fundal height. Point-of-care tests for heamoglobin and random glucose concentrations, a dried blood spot for diagnostic tests for malaria, and urine analysis for presence of protein, leukocytes and blood will be carried out.

### Monthly questionnaire

When participants reach 26 weeks gestational age, they will be visited monthly by the study team to administer a short questionnaire. This will include questions on recent illness (e.g. recent infections and medication use), the WHO-5 well-being Index assessment, subjective sleep disturbance using the PROMIS sleep disturbance form, food insecurity using the Food InsecurityExperience Scale from FAO and water insecurity using the abridged Water Insecurity Experience Scales.

### Study activities at 28 and 35 weeks of gestation

Each participant will be visited at their home by the field team and fitted with a wearable tracker device (Garmin vivosmart5™) that will be placed on the wrist of their dominant hand according to a standard operating procedure. This device will monitor heart rate, activity (steps and distance) and sleep. Once fitted, the participants will continue to perform their usual routine activities and will wear the device continuously for the next five days. All participants will be given a small portable device (Kestrel D2 drop) to measure temperature and humidity to attach to their clothing and wear in addition to the watch device. This will measure direct, localized environmental exposure for the week including information on indoor heat exposure.

In a random subset of participants, we will collect additional information on their environmental exposure and in-depth details on their daily activities and food and water intake. For the environmental monitoring, we will utilize small, portable indoor air pollution monitors to be placed in their homes and noise sensors to be placed outside their home on the facades by their bedrooms. During the course of their monitoring week they will be visited by field staff who will complete a task diary of their activities from 8am–6pm.

During the five days, participants will be visited regularly to ensure compliance with wearing the devices, check battery status of the devices, and administer the monthly questionnaire should this fall on the same week.

At the end of the five days every participant will undergo an ultrasound scan. This will include fetal growth parameters, fetal heart rate, placenta location, volume of amniotic fluid, and if available a Doppler examination of the umbilical artery. Point-of-care tests to include haemoglobin, glucose, dried-blood spot for malaria PCR and urine analysis, as well as blood pressure and basic clinical assessment will be carried out. Additionally, all participants will have a blood sample taken at the completion of the 28 week visit to test for circulating placental hormones and inflammatory markers at a later date.

### At delivery

Participants or midwives at the facilities will inform the study team when labour is established. Participants will be brought to the health facility by the study car (if they wish) and attended by government staff during the intrapartum period. Study staff will be present to collect data on duration of labour, medication given during labour, mode of delivery, complications of delivery, birth outcome and apgar score. Placental samples and cord blood will be taken within 30 mins of delivery of the placenta as detailed below. Neonatal anthropometric measurements will be taken at birth, to include birth weight, birth length, head circumference and infant sex, as well as a final maternal blood sample. On the occasion where participants deliver outside of the study hospital or the study team are unable to attend the birth, neonatal anthropometry will be collected up to 72 hours of birth and otherwise will be coded as missing.

### Placenta sampling

Each placenta will undergo gross examination and placental parameters, including placental weight, disc dimensions (length, weight and depth) and membrane features will be recorded including a photograph. Four samples will be taken from the maternal-facing portion of the placenta using a standardized biopsy quadrant method which takes into account the cord insertion site and is able to overcome heterogeneity in placental morphology and gene expression ^33^. These four samples will be taken mid-way from the cord insertion site to the peripheral edge of the placenta. Each 2cm cube biopsy from each quadrant will have ~0.5cm trimmed from the maternal facing edge and then will be divided into 3 equal parts that are either: 1) immediately snap frozen (all 4 quadrants snap frozen first); 2) put into RNA-later and then fixative; 3) and immediately fixed in paraformaldehyde for later molecular analyses ^34^.

### Neonatal-Behavioral Assessment Scale

Designated members of the study team will undergo training and certification in performing the neonatal-behavioral assessment scale (NBAS), organised by the Brazelton Centre UK. Each newborn will be assessed once at around 28 days using the NBAS Brazelton scale which includes 28 behavioral and 18 reflex responses. This assessment will be completed at the participant’s home or the clinic, in a quiet and safe place, mid-way between feeds. Assessment usually takes 20–30 minutes and is an interactive assessment covering how the baby responds to light and sound when asleep and how they follow faces and objects when awake.

### Final assessments

A final visit will occur when the infant is around 28 days old. Study staff members will perform a final clinical assessment and take two samples: a dried blood spot for infant molecular analyses. This will signify the end of the study for the participant.

## Laboratory processes

### Sample collection and processing

Trained study staff will collect a 14ml venous blood sample from every participant at the completion of 28 weeks gestation. The sample will be collected directly into the vacutainer tubes using the blood containing kit provided.

After delivery, every participant, where possible will have maternal blood, cord blood and placenta samples taken by a trained midwife or research clinician. These samples will be taken within 30 mins of the placenta being delivered. 13mLs of cord blood will be collected from the fetal vein to study whether metabolic substrates, hormones or cytokines levels released from the mother via the placenta may contribute to poor pregnancy outcomes in those exposed to heat stress.

### Sample storage

Whole blood samples from each participant will be used to prepare dried blood spots (DBS) of 10µl each on filter paper and stored for malaria PCR. Infant DBS at 28 days of life will also be collected onto filter paper and stored for later epigenetic analysis. Serum, plasma and a red blood cell pellet will be separated and stored at -80°C for both maternal and cord blood.

### Sample shipment

Samples will be shipped to University of Cambridge following MRCG@LSHTM SOPs in place. Where possible, samples will be shipped at standard temperature, but where necessary samples requiring -80°C will be shipped on dry ice. The samples shipped will be a subset of the full cohort and will be identified prior to shipping through environmental and clinical factors.

### Specific laboratory assessments

All laboratory protocols will be described in study specific SOPs.

Maternal blood at 28 weeks gestation will be assessed for the following maternal circulating placental hormones and inflammatory cytokine levels: pregnancy-associated plasma protein-A (PAPP-A), placenta growth factor (PLGF), tumour necrosis factor alpha (TNF alpha) and interleukin-6 (IL-6). As this is an area of rapidly growing interest, other relevant biomarkers may be identified which we would test on the maternal and cord blood (budget allowing).

Placenta examination: placental histology examination by robust unbiased stereology will include evaluation of villi density and maturation, syncytial knots, fibrosis, trophoblast, stroma, fetal capillaries, surface area and barrier to diffusion ^33, 34^. For the gene expression analysis, following RNA extraction, placental and infant samples will be analysed by RNA-sequencing to identify the genes which are differentially expressed with heat stress/strain. We will use gene ontology and pathway analyses (e.g. REVIGO, STRING and KEGG) to identify the cellular functions and signalling pathways that are enriched in the gene set that is differentially-expressed.

## Safety considerations

Risks associated directly with participating in this study are minimal. Participants will be identified when they attend the government-run antenatal services, and they will remain under the care of the free government service. In addition, they will be seen by study staff who will consist of trained nurse-midwives and research clinicians. At study visits, study staff will supplement the general antenatal care to ensure screening for anaemia, hypertension, proteinuria and urinary tract infections are completed. In addition, the study will cover the costs of any medication requirements for participants where these are not freely available by the government. Should at any time a participant fall unwell during the study period, they will have access to contact details for a study midwife who will arrange medical review and treatment if needed. Should the study midwife have any medical concerns that they feel they are unable to meet, then the government approved obstetric consultant will be contacted for clinical advice and the participant transferred/treated accordingly. Advice will also be sought when complications on ultrasound scan are identified, and transfer arranged as necessary.

At the time of commencement of labour, participants will be transferred to the hospital in a study vehicle if needed and study staff members will be present, although the participant will be under the care of the government-run facility.

## Data management

### Data quality assurance and monitoring

All study staff will be GCP certified and trained on the study protocol prior to the start of study activities. Data collection forms will be reviewed by the study coordinator and Principal Investigator (PI) for completeness. Regular meetings will be held with the study staff to discuss progress and any problems. Standardised protocols and SOPs will be followed to ensure quality control.

### Records

All participants will be allocated a unique identifying number at recruitment. Data generated by the Garmin devices will be downloaded from the devices using a suctom-developed Application Programming Interface at the end of the 5–7 days of wearing the device, linked to the unique identifying number and then reset. During the study, data will be collected on tablets using REDCap (Research Data Application Capture https://projectredcap.org/software/). Tablets will be synced at the field stations where stable internet connection allows transfer of encrypted data to the designated server.

All data will be backed up regularly by the IT department in accordance with MRCG@LSHTM standard protocols. The database is centrally stored, data is secure and encrypted. No personal identifiable information will be available in any shared or published document. Primary data outputs will be in csv format.

### Immediate and long-term use of the data

Data collected in the study will be analysed and prepared for publication in open-access peer-reviewed journals. We will comply with international standards and guidelines regarding open access of research data. Anonymized datasets will be made available on public open access sites (e.g. LSHTM’s datacompass) within two years of the completion of all aspects of the study. All study documents will be filed on secure, encrypted servers and stored for at least 10 years, in keeping with standard MRCG protocols.

## Data analysis and statistical considerations

### Accurately assign exposure

For heat stress exposure we will develop spatiotemporal statistical models. We will utilise machine learning to predict a set of heat stress indices to develop a 50×50-meter cell of the study area. The individual heat stress monitors worn by participants at 28 and 35 weeks of gestational age will be used as the ground truth or predictor variable. This will be used during model training to make predictions for the cohort of individual heat stress exposure throughout pregnancy. Stationary monitors, local weather station data, global gridded climate data sets and satellite imagery will be the target variables. The model will include additional environmental variables, such as, but not exclusively: Normalised Difference Vegetation Index (NDVI), land surface temperature, WBGT, UTCI. The model will be validated using a train and test split. We will then assess the spatial and temporal variation of each weather variable across the study region through maps and thermal drone imagery and identify high-risk neighbourhoods and contextual variables associated with high-low exposure and periods of years with higher/lower heat/heat stress or better/worse thermal comfort. Air pollution will be assigned from the stationary monitors which give hourly rolling average concentrations of PM1, PM2.5, PM 10 and AQI for the study regions.

### Sub-study details

In 60 participants we will estimate indoor air pollution and noise pollution. In addition to air pollution, residents of urban environments are often exposed to higher noise levels, which have shown to detrimentally affect sleep and brain function ^35, 36^. These participants will be randomly selected from those due their 28 or 35 week visit and will have additional monitors placed at their home. The Smart Citizen Kit 2.3™ will be used to monitor air pollution in the cooking area and the NSRT_mk4™ will be placed outside the bedroom window to monitor noise exposure. This subset of data will be analysed independently to the main study to give insights into the interaction between noise, indoor air pollution and heat exposure with maternal physiological measurements in this setting.

### Maternal physiology

Physiological heat strain will be defined using information from the wearable devices. We will measure the level of physiological heat strain through the estimation of the physiological strain index (PSI). It is a widely accepted metric for assessing physiological heat strain that goes from 0 (no strain) to 10 (very high strain). We will not have access to measured core temperature in our participants and therefore will use validated algorithms to compute heat strain from the non-invasive wearable device data ^37^. We will also evaluate physiological heat strain based on reported heat strain symptoms collected by field staff. We will apply the case time series design and perform linear, non-linear, and logistic mixed models to assess the association between exposure to heat and heat stress and both physiological heat strain and sleep quality as determined from the wearable devices. We will assess if the association was different according to baseline characteristics and gestational age through stratified analyses and interaction terms.

### Materno-placental blood flow

Materno-placental blood flow will be determined by the umbilical artery resistance index (a measure of umbilical artery blood velocity). This varies by gestational age and therefore the gestational-age adjusted z-score will be calculated and used in the analysis. The effect of acute heat exposure on the umbilical artery z-score will be assessed using a mixed model, non-linear regression model, with heat exposure defined by heat and heat stress in the preceding week (from the directly observed measurements) and including important covariates such as air pollution exposure and maternal heat strain (from the wearable device). Chronic heat stress on umbilical blood flow will be assessed by assigning heat stress exposure during the entire pregnancy to date.

### Pregnancy outcomes

Pregnancy exposure by trimester will be used to evaluate the impact of heat in pregnancy on birth outcomes, structural and transcriptomic changes in the placenta, and neonatal neurocognitive behavioural, molecular and microbiome changes. Birth outcomes will include fetal loss, preterm birth, low birth weight, small-for-gestational age and birth anthropometry adjusted for gestational age. The INTERGROWTH-21 reference will be used as reference and to determine gestational-age appropriate z-scores ^38^. Linear, non-linear, non-linear distributed lag models and logistic regression models will be used for these analyses.

## Quality assurance

Quality control of all study procedures will be assured by conducting all study procedures according to SOPs. An advisory monitoring board will be appointed and will meet regularly to assess progress, data reports and participant safety considerations.

## Expected outcomes of the study

This study will give valuable information on heat exposure of pregnant Gambian women, their physiological responses, placental function, sleep quality, birth outcomes and assessment of newborn neuro-behavior. This information can be directly utilized to develop interventions (i.e. identification of at risk individuals or exposure thresholds) and to evaluate interventions and adaptations to reduce heat exposure health impacts in pregnancy.

## Dissemination of results and publication policy

A high degree of community engagement is essential for this project to work. The community leaders will be invited to consider and discuss the project prior to commencement. At the end of the project a community event will be held to disseminate results to all those communities that participated in the study.

The advisory board will provide advice and oversight to help disseminate our findings to generate impactful outcomes. The results from the study will be prepared for publication in open access peer-reviewed journals as soon as possible on completion of the study. Results will also be presented at local and international conferences, public webinars, to key government organisations (e.g. Ministry of Health and Ministry of Environment) and at international events (such as COP).

## Ethics and consent

This study follows the international ethical codes as set out in the Declaration of Helsinki ( https://www.wma.net/policies-post/wma-declaration-of-helsinki-ethical-principles-for-medical-research-involving-human-subjects/). The study was reviewed and approved by the Gambian Government/Medical Research Council ethics committee (on the 19th Jan 2024) and by the London School of Hygiene and Tropical Medicine ethics committee (on the 30th Jan 2024; approval number: 29917). We also received approval from the Ministry of Health, Republic of the Gambia to undertake this study on the 8th March 2024. Written informed consent will be obtained from all participants. Participants will be identified by unique identification numbers in all data collection forms and databases. No identifiable data will be shared or published. Data is securely stored on MRCG/LSHTM servers and all data is held by MRCG/LSHTM.

## Data Availability

No data are associated with this article.
