# Supplementary material for: Study protocol for an observational cohort study of heat stress impacts in pregnancy in The Gambia, West Africa
Source: Wellcome Open Res. 2025 Feb 7;9:624. Originally published 2024 Oct 28. [Version 2] doi: 10.12688/wellcomeopenres.23172.2 (PMC11803196; doi:10.12688/wellcomeopenres.23172.2)
Supplement: Peer review history

# Reviewer report: Reviewer response for version 2

**DOI:** 10.21956/wellcomeopenres.26241.r118297

**Published:** 2025-02-24

**Authors:** Caitlin Wyrwoll

**Affiliations:** 1 School of Human Sciences, The University of Western Australia, Crawley, WA, Australia

I am happy with the changes and approve the article.

Is the study design appropriate for the research question?

Yes

Is the rationale for, and objectives of, the study clearly described?

Yes

Are sufficient details of the methods provided to allow replication by others?

No

Are the datasets clearly presented in a useable and accessible format?

Not applicable

Reviewer Expertise:

Reproductive physiology: environmental exposures in pregnancy.

I confirm that I have read this submission and believe that I have an appropriate level of expertise to confirm that it is of an acceptable scientific standard.

# Reviewer report: Reviewer response for version 1

**DOI:** 10.21956/wellcomeopenres.25517.r114245

**Published:** 2024-12-16

**Authors:** Zhiwei Xu

**Affiliations:** 1 Griffith University, Gold Coast, Australia

In this study protocol, Dr. Bonell and colleagues present the details of a prospective cohort study on the impacts of heat stress during pregnancy in Gambia. The proposed study is very interesting, and I enjoyed reading the protocol. I have several comments for the authors to consider:

**Main comments:**

 1. In Table 1, the authors provide detailed information used in the sample size calculation (n = 764). It would be helpful if they could provide the sources of evidence for the effect sizes they proposed in this table (e.g., 0.15, 0.2, and 0.14).

 2. It is great that the authors have considered potential loss to follow-up in the sample size calculation. To improve clarity, it would be helpful if they could provide details on the expected drop-out rate and explain why they anticipate this rate.

 3. I believe the authors may have previous experience interacting with government staff and pregnant women in the study setting through their past projects. If this is the case, could the authors please briefly mention their relevant experiences in the Methods section? Previous interactions with the target population would strengthen the practicality of participant recruitment.

 4. The authors propose validating the 50 x 50 m grid cell heat exposure data with data collected from individual heat stress monitors. However, it seems that the grid cell data represents outdoor heat exposure, while the individual monitor data likely represents indoor heat exposure. Although indoor heat exposure is often correlated with outdoor exposure, they are not necessarily the same. It would be helpful if the authors could provide more details on the data validation process.

**Minor comment:**

 Given that the authors plan to use wearable tracker devices to collect data on pregnant women’s heart rate, activity, and sleep, it would be worthwhile to consider establishing a reference group before initiating data collection. This would help better understand pregnant women’s interest in and capacity for interacting with digital technology in the study setting.

Is the study design appropriate for the research question?

Yes

Is the rationale for, and objectives of, the study clearly described?

Yes

Are sufficient details of the methods provided to allow replication by others?

Partly

Are the datasets clearly presented in a useable and accessible format?

Not applicable

Reviewer Expertise:

Heat epidemiology and biostatistics

I confirm that I have read this submission and believe that I have an appropriate level of expertise to confirm that it is of an acceptable scientific standard.

# Reviewer report: Reviewer response for version 1

**DOI:** 10.21956/wellcomeopenres.25517.r112341

**Published:** 2024-12-07

**Authors:** Pam Factor-Litvak

**Affiliations:** 1 Columbia University,, New York, USA

Minor comments:

 1. Birth outcome assessments: please standardize the timing of collection because, for example, birth weight can vary between delivery and 72 hours after birth.  Or, alternatively, collect these data at both times.

 2. Will the newborn anthropometrics be collected at the time of the NBAS assessment and at the final assessment at 28 days?

 3. The fecal swab for microbiota at 28 days is important.  Perhaps consider also getting a meconium swab as well.  In that way you may be able to determine changes in the microbiota related to heat stress. 

 4. I would suggest considering using distributed lag models to assess possible critical times of exposure to heat stress for the various outcomes.

Is the study design appropriate for the research question?

Yes

Is the rationale for, and objectives of, the study clearly described?

Yes

Are sufficient details of the methods provided to allow replication by others?

Yes

Are the datasets clearly presented in a useable and accessible format?

Not applicable

Reviewer Expertise:

Environmental epidemiology, including air pollution and climate, with a focus on outcomes over the life course.

I confirm that I have read this submission and believe that I have an appropriate level of expertise to confirm that it is of an acceptable scientific standard, however I have significant reservations, as outlined above.

# Response

**Published:** 2025-02-04

**Authors:** Ana Bonell

**Affiliations:** London School of Hygiene & Tropical Medicine, UK

**Minor comments:**

 1. Birth outcome assessments: please standardize the timing of collection because, for example, birth weight can vary between delivery and 72 hours after birth.  Or, alternatively, collect these data at both times.  

**Author response: **Thank you for this comment. We endeavour to capture all birth anthropometry at the time of delivery, but in some cases (if the delivery occurs outside of the hospitals we are working in or at home) then we may miss this. Only in those cases, do we then visit the participants to collect the anthropometry and birth details, and we have a window of 72 hours otherwise the data will be recorded as missing. All the anthropometric data is time and date stamped so we should be able to determine if there has been a delay in recording the data and manage the data accordingly. The following text has been changed to reflect this (section 3.9)   Neonatal anthropometric measurements will be taken at birth, to include birth weight, birth length, head circumference and infant sex, as well as a final maternal blood sample. On the occasion where participants deliver outside of the study hospital or the study team are unable to attend the birth, neonatal anthropometry will be collected up to 72 hours of birth and otherwise will be coded as missing.

 2. Will the newborn anthropometrics be collected at the time of the NBAS assessment and at the final assessment at 28 days?  

**Author response: **The NBAS assessment will take place at around 28 days, along with a final review of the infant, a brief clinical exam and a short survey. The newborn anthropometry will be completed within 72 hours of delivery, but mostly at the time of delivery and only in exceptional circumstances will this be delayed to 72 hours. Please see changed to text (section 3.11)   Each newborn will be assessed once at around 28 days using the NBAS Brazelton scale which includes 28 behavioral and 18 reflex responses. This assessment will be completed at the participant’s home or the clinic, in a quiet and safe place, mid-way between feeds. Assessment usually takes 20-30 minutes and is an interactive assessment covering how the baby responds to light and sound when asleep and how they follow faces and objects when awake.

 3. The fecal swab for microbiota at 28 days is important.  Perhaps consider also getting a meconium swab as well.  In that way you may be able to determine changes in the microbiota related to heat stress.   

**Author response: **Thank you for this comment. As indicated this is an important study and would require several other samples to allow a detailed and comprehensive analysis. Therefore after careful consideration of budget and man-power constraints we have dropped this aspect of the study. We have removed this completely from the study protocol and will hope that a different cohort will have the capacity to focus on this.

 4. I would suggest considering using distributed lag models to assess possible critical times of exposure to heat stress for the various outcomes.  

**Author response: **Thank you for this comment. We will certainly consider a distributed lag model – we have added this to the data analysis plan in section 7.5, see below text:   Linear, non-linear, non-linear distributed lag models and logistic regression models will be used for these analyses.

# Reviewer report: Reviewer response for version 1

**DOI:** 10.21956/wellcomeopenres.25517.r108580

**Published:** 2024-11-20

**Authors:** Caitlin Wyrwoll

**Affiliations:** 1 School of Human Sciences, The University of Western Australia, Crawley, WA, Australia

This is an extensive outline of the study which aims to create a whole picture of heat exposure experienced by pregnant women in a coastal and inland area of The Gambia. The outcomes of this study will be a valuable addition to the field. My major concern is that there is currently methodology context missing which needs to be added for clarity.

***Major comments***

- The assessment of air and noise pollution and other environmental factors such as food and water security are being conducted as a substudy. It is unclear how these factors will be used in the data analysis as a potential confounder of the effects of heat. This, and how it will be factored in to the study conclusions, needs to be clearer. 
- The methodology for the questions asked at enrolment and then monthly is unclear. Will standardised/validated questionnaires be used (eg. IWISE)? please provide more information on this. Given the Investigators are spending this time with the participants, it would be useful to also ask them their views on the impact of heat on pregnancy, as well as their opinions on solutions and needs to prevent the adverse impact of heat on pregnancy outcomes.
- Cord blood samples will be taken at birth from “fetal vein” (page 10). The vein in the umbilical cord is the vessel from the mum/placenta. Would an umbilical arterial cord sample be more appropriate to represent fetal metabolism/blood gas? 
- The placental sampling approach is unclear and it needs a standardised methodology detailed to ensure limited variability with the different people responsible for taking these samples. Why are samples being taken from the maternal-facing portion of the placenta? Normally samples are taken from the fetal-facing side as the site of the umbilical cord insertion can impact the establishment of quadrants for taking the samples. Will the samples be the complete depth of the placenta and in what order will the four samples be taken? Will there be further dissection of the samples so that the villous portion of the placenta is analysed using RNA seq? How will  collected samples be orientated to ensure the maternal and fetal aspects of the placenta are readily identified? Genes of interest via RNA seq of placental samples are not listed. The technique for the histological approach needs more detail - how are the syncytial knots identified using 2D stereology? Would 3D imaging approaches be more appropriate given it is likely that angiogenic pathways and fetal vascular branching patterns will have been impacted. 
- What wearable device will be used and how will the data be accessed and downloaded?
- How will the rectal swab be collected and what will be done to ensure limited contamination and how will they be stored? Please provide more comprehensive information on the microbiota analysis.

***Minor comments***

- Full stop missing after first paragraph of introduction (pg 4)
- Full stop/”and” missing in first sentence of “important potential confounders” paragraph. (pg 11)
- Reword second paragraph under data analysis and statistical considerations: maternal physiology. Very long sentence with lots of “ands”  (pg 11)
- mL not mls (pg 10)
- “Maternal hyperthermia increases the risk of normal embryonic and fetal development” – rephrase to indicate increasing the risk of negatively affecting development (pg 4)
- Some concluding sentences are spaced as a lone separate paragraph throughout – tag onto end of preceding paragraph

Is the study design appropriate for the research question?

Yes

Is the rationale for, and objectives of, the study clearly described?

Yes

Are sufficient details of the methods provided to allow replication by others?

No

Are the datasets clearly presented in a useable and accessible format?

Not applicable

Reviewer Expertise:

Reproductive physiology: environmental exposures in pregnancy.

I confirm that I have read this submission and believe that I have an appropriate level of expertise to confirm that it is of an acceptable scientific standard, however I have significant reservations, as outlined above.

# Response

**Published:** 2025-02-04

**Authors:** Ana Bonell

**Affiliations:** London School of Hygiene & Tropical Medicine, UK

***Major comments***

**Comment:** The assessment of air and noise pollution and other environmental factors such as food and water security are being conducted as a substudy. It is unclear how these factors will be used in the data analysis as a potential confounder of the effects of heat. This, and how it will be factored in to the study conclusions, needs to be clearer. 

**Author response:** Thank you for this valid comment. We have attempted to make our analysis plan clearer by expanding on the details of the main study as well as clarifying what the sub-study involves. Please see edited text below (found on section 7 of the protocol):

1. Accurately assign exposure

For heat stress exposure we will develop spatiotemporal statistical models. We will utilise machine learning to predict a set of heat stress indices to develop a 50x50-meter cell of the study area. The individual heat stress monitors worn by participants at 28 and 35 weeks of gestational age will be used as the ground truth or predictor variable. This will be used during model training to make predictions for the cohort of individual heat stress exposure throughout pregnancy. Stationary monitors, local weather station data, global gridded climate data sets and satellite imagery will be the target variables. The model will include additional environmental variables, such as, but not exclusively: Normalised Difference Vegetation Index (NDVI), land surface temperature, WBGT, UTCI. The model will be validated using a train and test split. We will then assess the spatial and temporal variation of each weather variable across the study region through maps and thermal drone imagery and identify high-risk neighbourhoods and contextual variables associated with high-low exposure and periods of years with higher/lower heat/heat stress or better/worse thermal comfort. Air pollution will be assigned from the stationary monitors which give hourly rolling average concentrations of PM1, PM 2.5, PM10 and AQI for the study regions. 7.2 Sub-study details: In 60 participants we will estimate indoor air pollution and noise pollution. In addition to air pollution, residents of urban environments are often exposed to higher noise levels, which have shown to detrimentally affect sleep and brain function. ^36,37^ These participants will be randomly selected from those due their 28 or 35 week visit and will have additional monitors placed at their home. The Smart Citizen Kit 2.3Ô will be used to monitor air pollution in the cooking area and the NSRT_mk4Ô will be placed outside the bedroom window to monitor noise exposure. This subset of data will be analysed independently to the main study to give insights into the interaction between noise, indoor air pollution and heat exposure with maternal physiological measurements in this setting.  

**Comment:**   The methodology for the questions asked at enrolment and then monthly is unclear. Will standardised/validated questionnaires be used (eg. IWISE)? please provide more information on this. Given the Investigators are spending this time with the participants, it would be useful to also ask them their views on the impact of heat on pregnancy, as well as their opinions on solutions and needs to prevent the adverse impact of heat on pregnancy outcomes.

**Author response: **The questions, where possible are based on validated questionnaires that have prior use at an international level. For well-being we used the WHO well-being Index: https://www.who.int/publications/m/item/WHO-UCN-MSD-MHE-2024.01#:~:text=The%20WHO%2D5%20is%20a,translated%20into%20over%2030%20languages. For food insecurity we used FAO’s Food Insecurity Experience Scale: https://openknowledge.fao.org/server/api/core/bitstreams/7dad7fe4-b4ec-4ffa-be41-ac7c32910c96/content For water security we used the abridged Water Insecurity Experiences (WISE) scales: https://www.ipr.northwestern.edu/wise-scales/ For sleep disturbance we used the PROMIS sleep disturbance short form: https://www.painconsortium.nih.gov/sites/default/files/documents/PROMIS_SF_v1.0_Sleep_Disturbance_4a.pdf See updated text below: This will include questions on recent illness (e.g. recent infections and medication use), the WHO-5 well-being Index assessment, subjective sleep disturbance using the PROMIS sleep disturbance form, food insecurity using the Food Insecurity Experience Scale from FAO and water insecurity using the abridged Water Insecurity Experience Scales.   Thank you for your comments on asking about views of impact of heat on pregnancy, solutions etc. We have planned this as a qualitative piece of work that will include in-depth interviews, focus groups discussions and workshops on solutions. This will be extensive and therefore is its own stand-alone study (although there will be overlap in recruitment and it will be undertaken in the same areas).

**Comment:** Cord blood samples will be taken at birth from “fetal vein” (page 10). The vein in the umbilical cord is the vessel from the mum/placenta. Would an umbilical arterial cord sample be more appropriate to represent fetal metabolism/blood gas? 

**Author response**: This is indeed true – that the arterial cord blood sample would be informative for assessing fetal metabolism. However we are interested in what the fetus is receiving from the mother via the placenta, which is why we are sampling the fetal vein. We are also particularly interested in metabolic substrate, hormone and cytokine levels – as we hypothesize a key role for impaired placental nutrient supply, reduced hormone production and increased release of inflammatory cytokines into the fetal circulation that contribute to poor pregnancy outcomes in heat stressed mothers. See additional text added (section 4.1): 13mL of cord blood will be collected from the fetal vein to study whether metabolic substrates, hormones or cytokines levels released from the mother via the placenta may contribute to poor pregnancy outcomes in those exposed to heat stress.

**Comment:** The placental sampling approach is unclear and it needs a standardised methodology detailed to ensure limited variability with the different people responsible for taking these samples. Why are samples being taken from the maternal-facing portion of the placenta? Normally samples are taken from the fetal-facing side as the site of the umbilical cord insertion can impact the establishment of quadrants for taking the samples. Will the samples be the complete depth of the placenta and in what order will the four samples be taken? Will there be further dissection of the samples so that the villous portion of the placenta is analysed using RNA seq? How will  collected samples be orientated to ensure the maternal and fetal aspects of the placenta are readily identified? Genes of interest via RNA seq of placental samples are not listed. The technique for the histological approach needs more detail - how are the syncytial knots identified using 2D stereology? Would 3D imaging approaches be more appropriate given it is likely that angiogenic pathways and fetal vascular branching patterns will have been impacted. 

**Author response:** We are using our established method for sampling the human placenta which takes into account the cord insertion site and is able to overcome heterogeneity in placental morphology and gene expression ( https://www.mdpi.com/2073-4409/12/7/1093). The exception to this published method, is that for our study, we are taking 1 instead of 2 samples per quadrant. These 4 samples are being taken mid-way from the cord insertion site to the peripheral edge of the placenta. We have intentionally chosen to sample from the maternal facing portion of the placenta, as that is the site of main materno-fetal substrate exchange. We obtain a 2cm cube biopsy from each quadrant, trim off ~0.5cm from the maternal facing edge and then divide that remaining villous sample into 0.5cm3 pieces that are immediately snap freeze, put into RNA-later and immersed in fixative (completing the snap freezing for all quadrants first, then RNA-later and then fixative). We are not taking a sample that has the complete depth of the placenta – and so there is no need to discriminate the fetal from the maternal facing portions of the placenta. Main genes of interest include those related to growth, angiogenesis/endothelial function, inflammation, nutrient transport, endocrine support of pregnancy, and heat/cellular stress ( https://pubmed.ncbi.nlm.nih.gov/37501633/). The method for quantifying syncytial knots can be found in our paper ( https://www.mdpi.com/2073-4409/12/7/1093). If we indeed find vascular changes in the placenta, in the future and with more resources, we would be very keen to employ 3D imaging. Please find edited text below (found in study protocol under section 3.10) 3.10 Placenta sampling Each placenta will undergo gross examination and placental parameters, including placental weight, disc dimensions (length, weight and depth) and membrane features will be recorded including a photograph. Four samples will be taken from the maternal-facing portion of the placenta using a standardized biopsy quadrant method which take into account the cord insertion site and is able to overcome heterogeneity in placental morphology and gene expression[AS1] . These four samples will be taken mid-way from the cord insertion site to the peripheral edge of the placenta. Each 2cm cube biopsy from each quadrant will have ~0.5cm trimmed from the maternal facing edge and then will be divided into 3 equal parts that are either: 1) immediately snap frozen (all 4 quadrants snap frozen first); 2) put into RNA-later and then fixative; 3) fixed in paraformaldehyde, for later analyses. ^34^  

**Comment:** What wearable device will be used and how will the data be accessed and downloaded?

**Author response:** Wearable device is the Garmin Vivosmart 5 tracker (section 3.8). Details of how the data will be accessed has been added to section 6.1 – see below:   All participants will be allocated a unique identifying number at recruitment. Data generated by the Garmin devices will be downloaded from the devices, using a custom-developed Application Programming Interface at the end of the 5-7 days of wearing the device, linked to the unique identifying number and then reset.

**Comment:** How will the rectal swab be collected and what will be done to ensure limited contamination and how will they be stored? Please provide more comprehensive information on the microbiota analysis.

**Author response: **Thank you for this comment. We have with careful consideration of budget and man-power constraints had to drop this aspect of the study. The microbiota analysis had significant time and cost implications which unfortunately the study is unable to deliver on. Therefore we have removed this completely from the study protocol and will hope that a different cohort will have the capacity to focus on this.

***Minor comments***

**Comment:** Full stop missing after first paragraph of introduction (pg 4)

**Author response: **added

**Comment:** Full stop/”and” missing in first sentence of “important potential confounders” paragraph. (pg 11)

**Author response: **Edited out in rewording as above.

**Comment:** Reword second paragraph under data analysis and statistical considerations: maternal physiology. Very long sentence with lots of “ands”  (pg 11)

**Author response: **Edited out in rewording as detailed above

**Comment:** mL not mls (pg 10)

**Author response: **changed

**Comment:** “Maternal hyperthermia increases the risk of normal embryonic and fetal development” – rephrase to indicate increasing the risk of negatively affecting development (pg 4)

**Author response: **Changed to: Maternal hyperthermia increases the risk of abnormal embryonic and fetal development.

**Comment:** ​​​​​​​Some concluding sentences are spaced as a lone separate paragraph throughout – tag onto end of preceding paragraph

**Author response: **changed
